# The Role of the Dysfunctional Akt-Related Pathway in Cancer: Establishment and Maintenance of a Malignant Cell Phenotype, Resistance to Therapy, and Future Strategies for Drug Development

**DOI:** 10.1155/2013/317186

**Published:** 2013-12-05

**Authors:** Gaetano Romano

**Affiliations:** Department of Biology, College of Science and Technology, Temple University, Bio Life Science Building, Suite 456, 1900 N. 12th Street, Philadelphia, PA 19122, USA

## Abstract

Akt serine/threonine kinases, or PKB, are key players in the regulation of a wide variety of cellular activities, such as growth, proliferation, protection from apoptotic injuries, control of DNA damage responses and genome stability, metabolism, migration, and angiogenesis. The Akt-related pathway responds to the stimulation mediated by growth factors, cytokines, hormones, and several nutrients. Akt is present in three isoforms: Akt1, Akt2, and Akt3, which may be alternatively named PKB**α**, PKB**β**, and PKB**γ**, respectively. The Akt isoforms are encoded on three diverse chromosomes and their biological functions are predominantly distinct. Deregulations in the Akt-related pathway were observed in many human maladies, including cancer, cardiopathies, neurological diseases, and type-2 diabetes. This review discusses the significance of the abnormal activities of the Akt axis in promoting and sustaining malignancies, along with the development of tumor cell populations that exhibit enhanced resistance to chemo- and/or radiotherapy. This occurrence may be responsible for the relapse of the disease, which is unfortunately very often related to fatal consequences in patients.

## 1. Introduction

Akt serine/threonine protein kinases are also termed PKB and constitute fundamental intracellular signaling systems for the regulation of an ample assortment of cellular and physiological activities, such as cell growth, proliferation, protection from apoptosis, modulation of DNA damage response and genome stability, motility, angiogenesis, and metabolism [1–7]. These Akt-mediated cellular functions are regulated by various types of external stimuli, which derive from the interaction of growth factors, hormones, cytokines, and nutrients with specific cellular receptors [1–7]. Some of the main hormones and growth factors that have the ability to stimulate the Akt axis comprise epidermal growth factor (EGF), insulin, insulin-like growth factor-I (IGF-I), vascular endothelial growth factor (VEGF), and nerve growth factor (NGF) [1–6]. Basically, the interaction between the external factors and the Akt axis occurs via ligand-cellular receptor binding, which, in turn, results in the transient Akt phosphorylation, with consequent temporary activation of the Akt intracellular signaling system. Overall, the Akt stimulation mediated by growth factors regulates cell cycle transition from G1/S to G2/M phase [1–7]. In addition, the Akt-related pathway comes into play in the orchestration of the DNA damage response and cellular genome stability [7]. Intracellular upstream effectors that activate the Akt-related pathway include phosphatidylinositol 3-kinase (PI3K) [8–10], LKB1 [11], and phosphatase and tensin homologue deleted on chromosome ten (PTEN) [12], whereas downstream regulators consist of mammalian target of rapamycin (mTOR) [13–15], eukaryotic initiation factor 4E (eIF4E) [16, 17], and tuberous sclerosis complex 2 (TSC2) [18–21]. Genetic analysis revealed that cellular protooncogenes encode Akt, eIF4E and the PI3K p85a regulatory subunit and p110 catalytic domain [1–10, 16, 17]. Instead, tumor suppressor genes encode TSC2, PTEN, and LKB1 [11, 12, 18–21].

**Figure 1:**
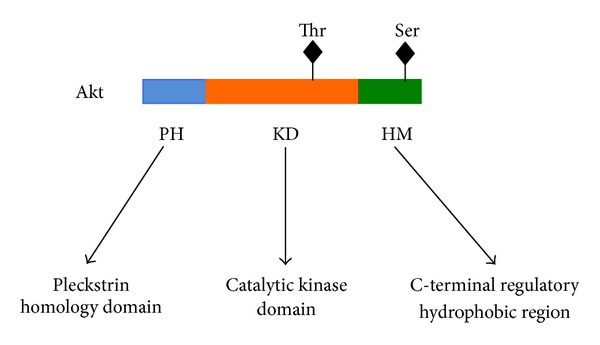
This figure illustrates the basic structure that is common among the three Akt isoforms (Akt1, Akt2, and Akt3). Each Akt isoform has three subdivisions: the Pleckstrin homology domain (PH), the catalytic kinase domain, and the C-terminal regulatory hydrophobic region (HM). In addition, the Akt activating threonine (Thr) and serine (Ser) residues are indicated in the figure. The coordinates of these two amino acid residues vary slightly among the three Akt isoforms and are listed in Table 1. The phosphorylation of these threonine and serine residues induces the activation of the Akt signaling system.

The Akt protein kinase family is present in three isoforms: Akt1, Akt2, and Akt3, which are also termed PKB*α*, PKB*β*, and PKB*γ*, respectively [1–5]. The three Akt isoforms belong to the class of AGC kinases [1–5]. Moreover, they are encoded on three distinct chromosomes, share a considerable homology, and contain three common structures: the N-terminal pleckstrin homology domain (PH), the catalytic kinase domain (KD), and the C-terminal regulatory hydrophobic region (Figure 1) [1–5]. The catalytic and regulatory domains are both critical for the biological actions mediated by Akt protein kinases and exhibit the maximum degree of homology among the three Akt isoforms [22, 23]. The PH domain binds lipid substrates, such as phosphatidylinositol (3,4) diphosphate (PIP2) and phosphatidylinositol (3,4,5) triphosphate (PIP3). The ATP binding site is situated approximately in the middle of the catalytic kinase domain, which has a substantial degree of homology with the other components of the AGC kinases family, such as p70 S6 kinase (S6K) and p90 ribosomal S6 kinase (RSK), protein kinase A (PKA) and protein kinase B (PKB). The hydrophobic regulatory moiety is a typical feature of the AGC kinases family [1–6, 22, 23]. The concomitant phosphorylation of threonine and serine residues is essential to optimize the kinase activity of the three Akt isoforms [1–5]. These threonine and serine residues are positioned in marginally different locations in Akt1, Akt2, and Akt3 (Table 1). For instance, the most essential regulatory amino acid residues are threonine 308 and serine 473 in Akt1, whereas the amino acid residues are threonine 309 and serine 474 in Akt2. In the case of Akt3, the regulatory amino acid residues are threonine 305 and serine 472 [1–5].

Normally, Akt1 and Akt2 are ubiquitously present in every tissue, while Akt3 expression is more circumscribed in terms of tissue distribution and exhibits a predominant expression in the central nervous system, heart, testis, kidneys, lungs, and skeletal muscles [24–26].

In recent years, a variety of studies conducted in Akt isoform-specific knockout mice unequivocally demonstrated that the biological functions of the three Akt isoforms are for the most part dissimilar from one another [2, 27–31]. For example, Akt1 is essential for cell survival, as Akt1-null cells are more susceptible to apoptosis than Akt1-positive cells and Akt1 knockout mice are substantially smaller than wild-type littermates [32, 33]. Instead, Akt2 has a more prevalent role in the regulation of glucose homeostasis, as Akt2 knockout mice exhibit higher incidence of a type-2 diabetes-like illness and primary cell cultures derived from these animals show evident ineffective glucose consumption [34, 35]. Akt3 has a more predominant purpose in postnatal brain development, as Akt3 knockout mice exhibit a median 25% reduction in brain weight and size, even though no major anatomical deformities were reported in this study, besides a considerable decrease of white matter fiber connections in the *corpus callosum* [36]. Another report demonstrated that Akt2 has the ability to enhance the resistance of rod photoreceptor cells to apoptotic injuries that may be caused by light-related stress, whereas the other two Akt isoforms lack this property [28]. These findings were observed in knockout mice models, which also showed that light-induced cell stress specifically activates Akt2 [28]. Intriguingly, Akt1 is essential to enhance cell survival for the majority of cells, except for light-induced cell stress in rod photoreceptor cells, which explicitly necessitate the activation of Akt2.

On these grounds, the three Akt isoforms exhibit differential biological characteristics and kinase activities, which are in function of the cellular context. In addition, a defective and less active Akt-related pathway does not provide an efficient protection from apoptotic injuries, which may become a contributing factor in the pathogenesis and/or clinical progression of several human maladies, such as neurodegenerative diseases [37–41], illnesses of the cardiovascular system [42–45], and type-2 diabetes [33, 34, 46]. Conversely, the overexpression and/or constitutive enhanced activity of the Akt-related pathway were observed in a wide variety of human tumors [1, 2, 22, 23, 30, 47–55]. This paper discusses the implications of deregulations in the Akt signaling system that were reported in different types of cancer.

## 2. Aberrant Akt-Related Pathways in Carcinogenesis and Progression of the Disease

Carcinogenesis is a multistep process that depends on certain environmental factors and involves a series of genetic and epigenetic mutations, which, in turn, may result in the activation of cellular oncogenes and/or silencing of tumor suppressor genes [6, 56–72]. One of the hallmarks of the establishment and maintenance of a transformed cell phenotype is the overexpression and/or constitutive enhanced activity of the Akt-related pathway, as clearly indicated by several lines of investigation [1–5, 73–77]. As already mentioned, the Akt intracellular signaling system is a main performer for the preservation of the overall control of cellular biology [1–6]. This control necessitates a steady equilibrium between the activities of cellular tumor suppressor factors and protooncogenes within the Akt pathway [1–18]. If for some reason the balance should fail, the role of the Akt axis-associated protooncogenes tends to prevail and, consequently, cause the constitutive enhanced activation and/or overexpression of Akt-related factors, which may contribute to the establishment and/or maintenance of a malignant cell phenotype [1, 2, 22, 23, 30, 47–53, 73–77]. For example, a defective PTEN expression is very likely associated with an enhanced activity of the Akt axis, which is recurrently reported in many types of tumors [54, 61].

As anticipated, physiological levels of Akt activity take part in the regulation of DNA damage response and cellular genome stability [7]. However, constitutive enhanced levels of Akt activity may obstruct both ATR/Chk1 signaling and homologous recombination repair (HRR), either by direct phosphorylation of Chk1 and/or DNA topoisomerase 2-binding protein 1 (TopBP1) or via prevention of assembly to the sites of DNA damage of double-strand break (DSB) resection factors, such as breast cancer susceptibility gene 1 (Brca1), replication protein A (RPA), and Rad51 [7, 78–80]. Thus, high levels of Akt activity may result in genome instability among malignant cells because of the loss of checkpoints and/or impairment of HRR functions [7].

The protooncogene TCL1 boosts the stimulation of the Akt axis activity through binding to the Akt PH domain [22]. Under normal physiological conditions, TCL1 expression is confined to cell populations of the immune system, during the early stages of development [22]. The increment of TCL1 expression levels in somatic cells is correlated with aberrant Akt kinase activity, as reported in different types of hematological malignancies and seminoma [22, 55]. Moreover, TCL1 mediates Akt nuclear translocation [81]. The biological functions of nuclear Akt are currently under investigation [81, 82]. It has been proposed that the presence of Akt in the nucleus is instrumental in inhibiting apoptosis, by blocking the caspase-activated deoxyribonuclease [83].

An early study showed that Akt2 overexpression transformed mouse fibroblast NIH/3T3 cells [84], whereas another report indicated that Akt2 overexpression increased substantially metastatic features and invasion both in human breast cancer and human ovarian cell lines [85]. Conversely, Akt1 and Akt3 overexpression failed to reproduce the effects that were observed for Akt2 overexpression in the previously mentioned human tumor cell lines [85]. This is a further evidence that accounts for the nonredundancy of the three Akt isoforms.

Some studies showed an involvement of aberrant PI3K/Akt3 activity in human melanoma [50, 86]. For instance, 70% of biopsies derived from patients with melanoma exhibited abnormal activities in the PI3K/Akt3-related signaling system [50]. A subsequent report showed that an enhanced PI3K/Akt3 pathway activity is one of the main contributors in the genesis of melanoma [86]. Moreover, several other studies supported the implication of the deregulated PI3K/Akt pathway in the development and/or clinical progression of melanoma [87–91].

Elevated Akt1 expression levels were observed in human cancers of the gastric system [92], thyroid [23], and breast [93]. Similarly, estrogen receptor-negative breast cancer and androgen-independent prostate cancer lines exhibited a remarkable overexpression of Akt3 mRNA [94]. In this respect, several other reports showed the involvement of the hyperactive Akt signaling system in human tumors of the breast [67, 95–101] and prostate [61, 102–106]. Furthermore, increased levels of Akt2 expression were reported among the following human tumors: gliomas [107, 108], colorectal cancer [109], hepatocellular carcinoma [110], ovarian tumors [26], and pancreatic malignancies [111, 112].

In addition to enhanced levels of Akt expression, a number of Akt activating mutations were reported in various types of human cancers. For instance, a transforming point mutation that changes a single glutamic acid to lysine at amino acid residue 17 (E17K) within the PH domain confers a continuous state of activation in Akt1 [113]. This somatic point mutation was identified in human breast, ovarian, and colorectal tumors [113]. Intriguingly, the E17K point mutation was absent in Akt2 and Akt3 in the previously mentioned tumors [113], although an analogous point mutation in the Akt3 PH domain was found in human melanoma [114].

In most cases, point mutations that cause the constitutive activation of the Akt axis involve the genetic modification of the PI3K p110 catalytic subunit (PI3KCA) [115–123]. Such PI3KCA mutations were observed in a wide variety of human malignancies [121, 123]. Some of such human malignancies include cancers of the breast [115, 116, 119], gastric system [117], colorectal tract [120], oral cavity [118], and thyroid [122].

A deregulated Akt activity is among the main factors that are implicated in the establishment of a malignant phenotype and/or progression of the clinical course of the disease [1–6, 22, 23, 47–53]. On these grounds, the Akt-related pathway may be considered a suitable target for cancer therapy [52, 55, 57, 79, 124–126]. However, the inhibition of the Akt axis is one of the requirements for enhanced cell motility [127–132]. In fact, the Akt signaling system suppresses the activity of the nuclear factor of activated T cells (NFAT) [127–131], which is a transcription factor that increases both cell motility and invasion in different kinds of malignancies [127–135]. Most likely, the Akt-induced inhibition of NFAT activity occurs through the Akt-mediated stimulation of the E3 ubiquitin-protein ligase Mdm2, which, in turn, promotes the degradation of NFAT [132]. Thus, the pharmacological inhibition of the Akt-related pathway in cancer therapy might unexpectedly become a contributing factor for the dissemination of cancer metastases [132]. Indeed, this is a very important aspect that should be taken under consideration in the planning of various therapeutic strategies for the treatment of malignancies in patients.

## 3. Abnormal Akt-Related Pathways in Resistance to Cancer Therapy

**Figure 2:**
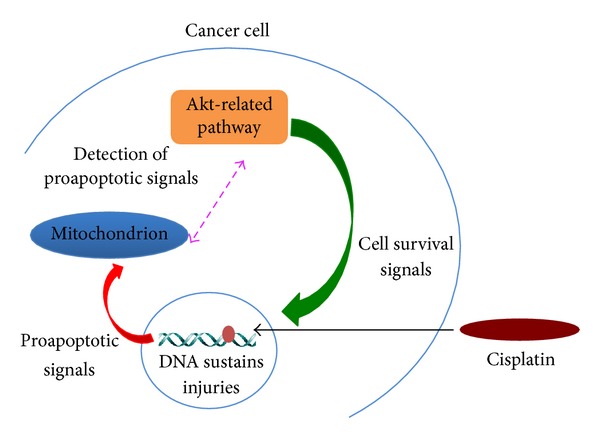
Basic off-target mechanism of Akt-induced malignant cell survival in response to cisplatin treatment.

Undoubtedly, the development of malignant cells with enhanced resistance to chemo- and/or radiotherapy is one of the most pressing issues for the field of oncology [62, 136–146]. The onset of cancer cell variants with increased resistance to therapy may cause the relapse of the illness, which is often associated with fatal consequences in patients [62, 136–146]. In this respect, an abundant number of reports persuasively confirmed that a deregulated Akt pathway is a key element for the generation of tumor cells with increased resistance to chemo- and/or radiotherapy [7, 136, 141, 143, 147–160]. For instance, the Akt-related pathway is one of the main factors that may intervene in the development of increased resistance to cis-diamminedichloroplatinum (II) therapy [141, 158, 159]. The anticancer compound cis-diamminedichloroplatinum (II) is more commonly known either as cisplatin or CDDP [141, 158, 159, 161] and has been utilized for the treatment of several types of solid tumors, such as ovarian, testicular, head and neck, lung, colorectal, and bladder cancers [141, 160–164]. Cisplatin-mediated suppression of tumor growth occurs through various types of mechanisms [141]. The best-characterized and also predominant mechanism of cisplatin anticancer action consists of producing lesions within the cancer cell genome [141, 165–167], which are followed by the intervention of the DNA damage response system and mitochondrial apoptosis [141, 168, 169]. Specifically, the Akt-related pathway confers resistance to malignant cells against cisplatin treatment through a so-called off-target resistance mechanism, which may be induced by intracellular signaling systems that are not directly affected by cisplatin and come into play in the attempt to counterbalance the cisplatin-derived lethal effects in target cells (Figure 2) [141, 170, 171]. Generally, the Akt-mediated off-target resistance to cisplatin takes place in two stages. Initially, the PI3K/Akt signaling system is maintained at a baseline activity [141]. At this stage, there is an increase of cyclin-dependent kinase inhibitor 1A (CDKN1A) expression levels within the cell nucleus [171]. CDKN1A is also termed either p21^Cip1^ or p21^Waf1^ [171]. During this period, the cisplatin-injured malignant cell may take advantage of a temporary CDKN1A-induced cell cycle arrest to try to repair the damaged genomic DNA [141, 171]. In a second stage, however, survived malignant cells must resume the proliferation program [141]. This occurs through a subsequent increment of PI3K/Akt activity, which, in turn, is responsible for the nuclear rejection of CDKN1A [141, 171, 172]. Once CDKN1A is outside the cell nucleus, it can no longer impose a cell cycle arrest and, therefore, malignant cells recommence to proliferate [141]. A hyperactive PI3K/Akt signaling system is one of the contributing factors that are also responsible for the development of cancer cells with increased resistance to a broad spectrum of chemotherapeutics [136–194] and radiotherapy [143–150, 155–157]. Some of the anticancer drugs that become clinically ineffective comprise paclitaxel [171, 173–180], doxorubicin [180–182], gefitinib [152, 183–187], imatinib [186, 188–192], and flavopiridol [193, 194]. The clinical and/or preclinical studies on the Akt pathway-mediated enhanced resistance to chemo- and/or radiotherapy were conducted on several types of human hematological tumors [195–198] and human epithelial malignancies [143, 147–160]. The latters included cancers of the brain, breast, ovaries, testicles, bladder, prostate, lung, colorectal tract, pancreas, and head and neck [143, 147–160].

In recent years, a number of PI3K/Akt/mTOR inhibitors have been developed for the treatment of different types of tumors [199–201]. Some PI3K/Akt/mTOR inhibitors comprise rapamycin, sirolimus, metformin, everolimus, and temsirolimus [199–201]. Although the PI3K/Akt/mTOR axis is a promising target for the treatment of cancer, randomized phase III clinical trials reported suboptimal beneficial therapeutic effects in patients [199–201]. Exceedingly high levels of toxicity were unfortunately observed in various clinical trials [199–201]. Moreover, the effects of the inhibition of the PI3K/Akt/mTOR pathway can be circumvented in cancer cells through the Raf/MEK/ERK signaling system [200], which may protect malignant cells from drug-induced proapoptotic injuries and, therefore, produce chemoresistant cancer cells variants [200].

In addition, the field of oncology is currently addressing the function of rare subpopulation of cancer cells with stem cell-like properties, or cancer stem cells, in the process of carcinogenesis, spreading of metastasis, regeneration of the tumor mass, and development of malignant cells with enhanced resistance to chemo and/or radiotherapy [152, 202–211]. Such rare subpopulations of malignant cells with stem cell-like properties express the surface marker CD133 (or Prominin-1), which renders possible their identification in neoplastic tissues [202, 203, 212–221]. The efficient detection of CD133 expression in malignant tissues might assume a considerable prognostic importance [214]. According to the so-called cancer stem cell hypothesis, only specialized subsets of malignant cells with stem cell-like features have the ability to originate and maintain a malignancy [202–211]. Moreover, cancer stem cells are more resistant to toxic agents and radiations than other tumor cells [154, 202–211]. Therefore, anticancer therapeutics eliminate most of the malignant cells, but some cancer stem cells might be able to survive and, eventually, they reconstitute the tumor mass with cancer cell populations that are more resistant to chemo- and/or radiotherapy [154, 202–221]. Of course, the Akt-related pathway plays a strategic role also in the biology of cancer stem cells, as convincingly demonstrated by several reports [154, 210, 222–226]. Indeed, the inhibition of the canonical Akt-related cell survival pathway constitutes a highly critical target for cancer therapy.

A study has recently shown that flavopiridol triggered a considerable Akt-Ser473 phosphorylation in human glioblastoma T98G cell line [194]. In contrast, as expected, flavopiridol treatment caused a reduction of Akt-Ser473 phosphorylation in human glioblastoma U87MG cell line and in human prostate cancer PC3 cell line [194]. As already discussed, Akt-Ser473 phosphorylation is a characteristic of the Akt-related pathway activation, which, in turn, may protect cells from apoptotic injuries [1–5]. Flavopiridol is a pan-inhibitor of cyclin-dependent kinases and has been used in several clinical trials for the treatment of patients with various kinds of malignancies, albeit with modest therapeutic efficacy [57, 227–230]. The use of flavopiridol is supposed to impair the cellular signaling systems for protection from apoptosis and survival [57, 227–230]. However, the previously mentioned study on human glioblastoma T98G cell line indicates that flavopiridol might paradoxically play a relevant role in the production of tumor cell variants with enhanced resistance to chemotherapy, through increased activation of the Akt-related pathway [57, 194]. For this reason, various anticancer drugs should be screened to assess whether or not they may incidentally induce the increment of Akt-Ser473 phosphorylation in different types of human tumor cells [194].

Interestingly, it was also reported that a deregulated Akt axis has the ability to confer radioresistance to malignant cells by orchestrating DNA repair through nonhomologous end joining (NHH) [7]. In this regard, a group of investigators observed a substantial *γ*-radiation-induced increment of Akt-Ser473 phosphorylation in a variety of human glioblastoma cell lines, such as U87MG, MO59J, and LN-18 [231].

Investigations are currently underway to determine the mechanisms of flavopiridol and/or *γ*-radiation-induced enhancement of Akt-Ser473 phosphorylation in human glioblastoma cell lines. In fact, a better understanding of these mechanisms may lead to the identification of novel therapeutic targets, which can be eventually suppressed with new drug formulations, in order to prevent the constitution of cancer cell variants that are more resilient to chemo- and/or radiotherapy.

## 4. Conclusion

Undeniably, a deregulated Akt pathway is an important factor in the establishment and/or maintenance of a malignant cell phenotype. Moreover, a constitutively activated Akt axis is involved in the generation of tumor cell variants with enhanced resistance to chemotherapeutic agents and/or radiotherapy.

On one hand, an abnormal Akt-related pathway is a very promising target to implement therapeutic approaches for the treatment of different types of cancer. On the other hand, the repression of the deregulated Akt signaling system, *per se*, does not seem to be sufficient for an effective therapy and may pose a number of collateral issues. For instance, a drug-induced inhibition of the Akt activity in malignant cells may unexpectedly contribute to the formation and/or dissemination of cancer metastases [127–132]. Another quite unforeseen side effect of the Akt pharmacological targeting is related to the flavopiridol-induced increment of Akt-Ser473 phosphorylation in human T98G glioblastoma cell line [194]. In addition, an increased Akt-Ser473 phosphorylation was observed following *γ*-irradiation of a panel of human glioblastoma cell lines [231]. All of these findings, taken together, suggest the pursuit of combinational therapeutic approaches for the treatment of different types of cancer [232–238], in order to prevent as much as possible treatment-related side effects that may paradoxically contribute to the spreading of metastases and/or to the generation of cancer cell variants with higher resistance to therapeutic interventions.

## Figures and Tables

**Table 1:** Coordinates of the Akt activating threonine (Thr) and serine (Ser) residues among the three Akt isoforms.

Akt isoform	Position of Akt activating Thr residue	Position of Akt activating Ser residue
Akt1	308	473
Akt2	309	474
Akt3	305	472
